# Supplementary material for: All-at-once RNA folding with 3D motif prediction framed by evolutionary information
Source: Nat Methods. 2025 Oct 3;22(10):2094–106. doi: 10.1038/s41592-025-02833-w (PMC12510878; doi:10.1038/s41592-025-02833-w)
Supplement: Supplementary file 3 — Source code to run CaCoFold-R3D. Data and results. [file 41592_2025_2833_MOESM3_ESM.gz › supplemental_material/Figures/Figure_5.pdf]

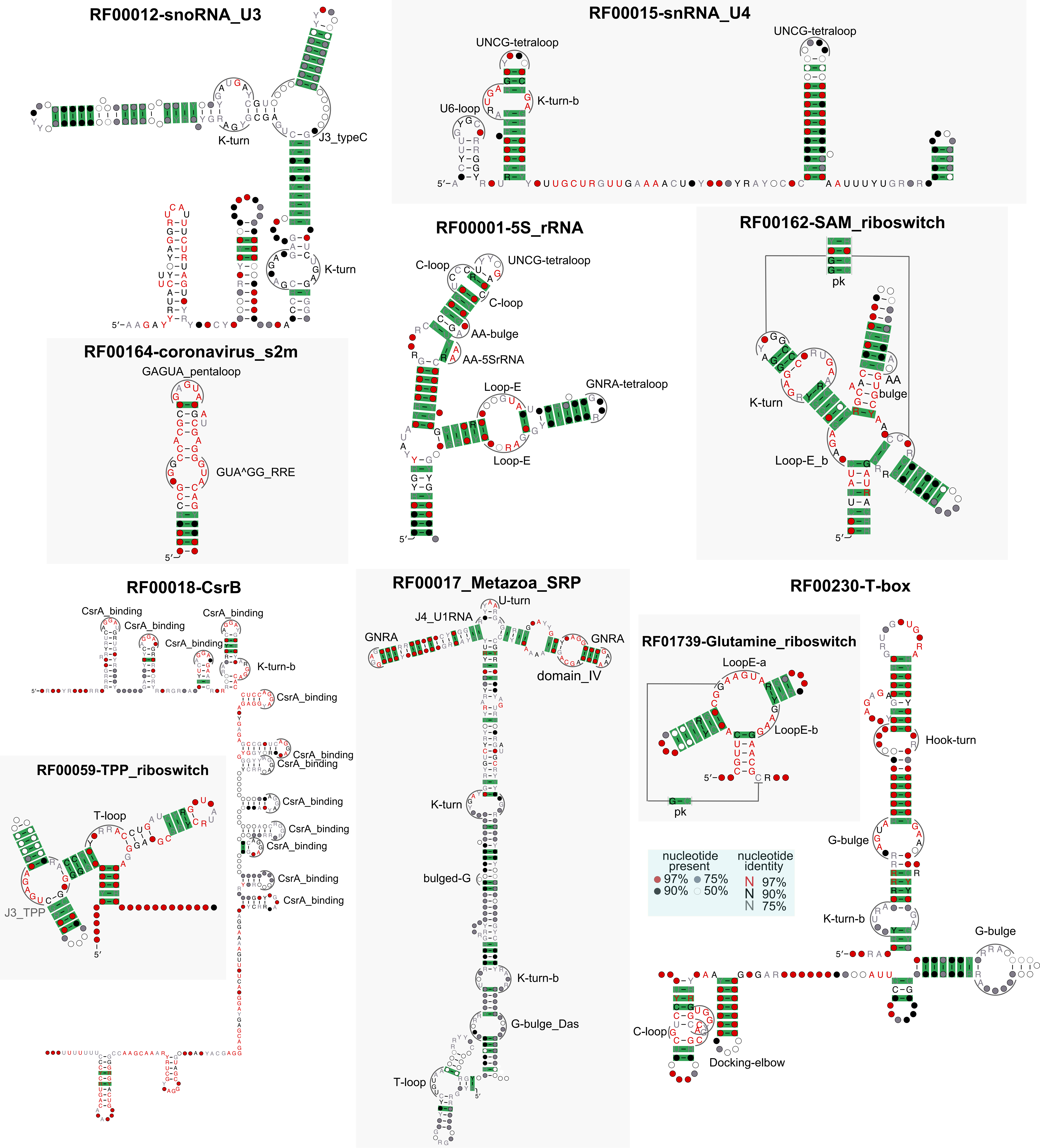
RF00012-snoRNA_U3
K-turn
J3_typeC
K-turn
RF00015-snRNA_U4
UNCG-tetraloop
K-turn-b
U6-loop
UNCG-tetraloop
RF00164-coronavirus_s2m
GAGUA_pentaloop
GUA^GG_RRE
RF00001-5S_rRNA
C-loop
UNCG-tetraloop
C-loop
AA-bulge
AA-5SrRNA
Loop-E
GNRA-tetraloop
Loop-E
RF00162-SAM_riboswitch
pk
K-turn
AA
bulge
Loop-E_b
RF00018-CsrB
CsrA_binding
CsrA_binding
CsrA_binding
CsrA_binding
K-turn-b
CsrA_binding
CsrA_binding
CsrA_binding
CsrA_binding
CsrA_binding
CsrA_binding
CsrA_binding
CsrA_binding
RF00059-TPP_riboswitch
T-loop
J3_TPP
RF00017_Metazoa_SRP
U-turn
J4_U1RNA
GNRA
GNRA
domain_IV
K-turn
bulged-G
K-turn-b
G-bulge_Das
T-loop
RF00230-T-box
RF01739-Glutamine_riboswitch
LoopE-a
LoopE-b
pk
Hook-turn
G-bulge
K-turn-b
G-bulge
C-loop
Docking-elbow
nucleotide present
97%
75%
90%
50%
nucleotide identity
N 97%
N 90%
N 75%
